# Triglyceride-Glucose Index Linked to In-Hospital Mortality in Critically Ill Patients with Heart Disease

**DOI:** 10.31083/j.rcm2308263

**Published:** 2022-07-21

**Authors:** Guangyao Zhai, Jianlong Wang, Yuyang Liu, Yujie Zhou

**Affiliations:** ^1^Cardiology Department, Beijing AnZhen Hospital: Capital Medical University Affiliated Anzhen Hospital, 100089 Beijing, China

**Keywords:** insulin resistance, TyG index, critically ill, heart disease, in-hospital mortality

## Abstract

**Background:**

As an alternative method to evaluate insulin resistance (IR), triglyceride-glucose index (TyG) was shown to be related to the severity and prognosis of cardiovascular diseases. The main aim of this study was to explore the association between TyG and in-hospital mortality in critically ill patients with heart disease.

**Method:**

The calculation method of TyG has been confirmed in previous report: Ln [fasting TGs (mg/dL) × FBG (mg/dL)/2]. All patients were divided into four different categories according to TyG quartiles. Primary outcome was in-hospital mortality. Binary logistic regression analysis was performed to determine the independent effect of TyG.

**Result:**

4839 critically ill patients with heart disease were involved. The overall mortality was 8.53 cases per 100 idviduals. In-hospital mortality increased as TyG quartiles increased (Quartile 4 vs Quartile 1: 12.1 vs 5.3, *p *< 0.001). Even after adjusting for confounding variables, TyG was still independently associated with the increased risk of in-hospital mortality in critically ill patients with heart disease (Quartile 4 vs Quartile 1: OR (95% CI): 1.83 (1.27, 2.64), *p *< 0.001, P for trend <0.001). In the subgroup analysis, we failed to observe the association between increased TyG and the risk of mortality in patients complicated by diabetes. In addition, as TyG quartiles increased, the length of intensive care unit (ICU) stay was prolonged (Quartile 4 vs Quartile 1: 2.3 (1.3, 4.9) vs 2.1 (1.3, 3.8), *p* = 0.007). And the significant interactions were not found in most subgroups.

**Conclusions:**

TyG was independently correlated with in-hospital mortality in critically ill patients with heart disease.

## 1. Introduction

In contemporary society, cardiovascular disease (CVD) is still the leading cause of morbidity and mortality worldwide. Especially, in patients with severe CVD, the mortality was greatly increased [1, 2]. In order to reduce the mortality of serious CVD patients, coronary artery care unit (CCU) and cardiac intensive care unit (CICU) came into being. After decades of development, CCU and CICU eventually focused on the management of patients with severe CVD which needed meticulous care and targeted treatment [3, 4]. Nowadays, the status of CCU and CICU are increasingly important and a variety of studies were performed to explore how to predict and improve prognosis of patients. As for clinicians, easily accessible and reliable prognostic indicators for critically ill patients with heart disease are always welcomed, especially, in patients with severe CVD, the mortality was greatly increased.

Type 2 diabetes mellitus (T2DM) has been widely proven to be one of the most significant risk factors for CVD [5]. The key mechanism of T2DM is insulin resistance (IR), which has been shown to be closely associated with the development of CVD and atherosclerosis [6, 7, 8, 9]. However, as the gold standard test for IR, the hyperinsulinaemic-euglycaemic clamp is time-consuming, expensive and complex [10], the triglyceride-glucose index (TyG index) is an alternative method, which evaluates IR by using the levels of glycemia (mg/dL) and fasting triglycerides (TG) (mg/dL) [11]. Studies have indicated that TyG index was associated positively with T2DM risk [12, 13, 14, 15]. Notably, previous studies have manifested that TyG index was related to the increased risk of worse outcomes in patients with CVD. Zhao *et al*. [16] recently demonstrated that TyG index had a prognostic role in patients with T2DM and non‑ST‑segment elevation acute coronary syndrome (NSTE-ACS) undergoing percutaneous coronary intervention (PCI). In addition, high TyG index was also proved to be associated with increased incidence of CVD events in healthy Caucasian and China participants [17, 18, 19]. Nevertheless, to the best of our knowledge, no research has demonstrated the effect of TyG index in patients with severe CVD. Thus, our main objective in this study was to investigate the relationship between TyG index and in-hospital mortality of critically ill patients with heart disease.

## 2. Method

### 2.1 Population Selection Criteria

**Fig. 1.**
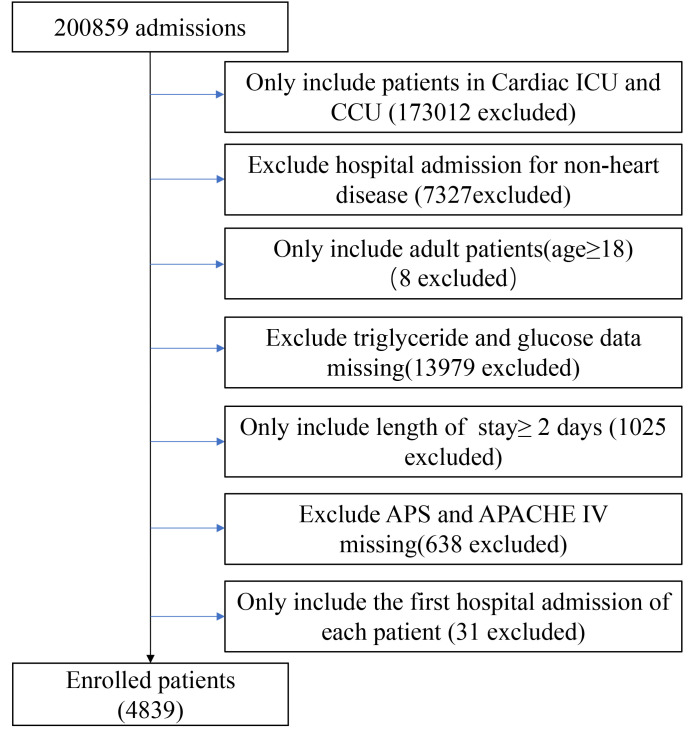
**Flow chart of study population**. Abbreviation: CCU, coronary artery care unit; CICU, cardiac intensive care unit.

The research objects were selected from CCU and CICU patients in eICU Collaborative Research Database [20]. Adult patients (≥18 years) hospitalized for more than 2 days at their first admission were available. Exclusion criteria are as follows: (1) hospital admission for non-heart disease; (2) triglyceride and glucose data missing; (3) acute physiology score (APS) and Acute Physiology and Chronic Health Evaluation IV (APACHE IV) data missing. A total of 4839 patients were included (Fig. 1).

### 2.2 Data Extraction

The original data for this study was from eICU Collaborative Research Database (https://doi.org/10.13026/C2WM1R) [20]. We passed the Protecting Human Research Participants exam to get access to the database (certificate number: 9728458).

Following data were collected: demographics, vital signs, body mass index, diagnoses and comorbidities, laboratory parameters, medication use, acute physiology score (APS) and Acute Physiology and Chronic Health Evaluation IV (APACHE IV) [21]. All data were extracted using Structured Query Language. Details was available in the **Supplementary Material** named “Data extraction”.

TyG index was obtained by the formula Ln [fasting triglycerides (mg/dL) × fasting glucose (mg/dL)/2]. Fasting triglycerides and fasting glucose were obtained by the first blood test after admission to ICU. The data of fasting triglycerides and fasting glucose were extracted using the “triglycerides” and “glucose” fields.

### 2.3 Grouping and Outcomes

Depending on the TyG quartiles, all enrolled patients were divided into four different categories. The primary outcome was in-hospital mortality. Secondary outcomes were length of intensive care unit (ICU) stay and length of hospital stay.

### 2.4 Statistical Analysis

Normally distributed continuous variables were expressed as mean ± standard deviation (SD) and compared between groups using analysis of variance. Skewed data were expressed as median and interquartile range (IQR) and compared using Kruskal–Wallis test. Categorical variables were expressed as number (percentage) and identified significant heterogeneity in the frequencies using Chi-square test.

Binary logistic regression analysis was used to identify the independent relationship between TyG and in-hospital mortality and the results were expressed as odds ratio (OR) and 95% confidence interval (CI). P for trend was calculated. Covariates were selected by statistical analysis and clinical doubt to modulate the outcome. Local weighted regression (Lowess) was used to plot the curve in line with overall trend, which described the probability of mortality predictded by TyG in raw calculations without adjustment for other covariates. Receiver-operating characteristic (ROC) curve was applied to evaluate the sensitivity and specificity of TyG. DeLong test was applied to compare the area under the curve (AUC) of different parameters. Subgroup analysis was used to determine the correlation between TyG and in-hospital mortality in different subgroups, P for interaction was calculated. A two-tailed *p* value < 0.05 was considered statistically significant. Stata V.15.1 (Statistical Analysis System, Raleigh, North Carolina, the United States) and MedCalc version 17 (MedCalc Software, Mariakerke, Belgium) were used to perform the data analysis.

## 3. Result

### 3.1 Subjects and Baseline Characteristics

4839 patients were analyzed (Fig. 1). According to TyG quartiles, all patients were divided into four groups: TyG <8.51 (n = 1201), 8.51 ≤ TyG < 8.92 (n = 1221), 8.92 ≤ TyG < 9.37 (n = 1214), TyG ≥9.37 (n = 1203). Table 1 showed the characteristics of different TyG groups. Patients with high TyG levels had the following characteristics: elder, Caucasian, higher blood pressure and higher body mass index. Furthermore, patients in higher PLR quartiles also tended to present more diagnoses and comorbidities of coronary artery disease, ST-elevation myocardial infarction (STEMI), acute coronary syndrome, non-ST-elevation myocardial infarction (NSTEMI), cardiac arrest, shock, respiratory failure, diabetes, chronic kidney disease, acute kidney injury, sepsis whereas less congestive heart failure, cardiomyopathy, valve disease, arrhythmias, bradycardia, atrial fibrillation, chronic obstructive pulmonary disease (COPD). Besides, Table 1 indicated that as TyG quartiles increased, white blood cell, lymphocyte percentage, red blood cell, hemoglobin, hematocrit, platelet, glucose, triglyceride, creatinine, blood nitrogen urea, potassium values tended to increase, while monocyte and neutrophil percentage, sodium values tended to decrease. There was no statistically significant difference in administration of medication among the TyG categories. Of note, patients with higher TyG index had significantly higher APS score which was used to evaluate the severity of ICU patients and predict their prognosis (Table 1).

**Table 1.** **Characteristics of patients stratified by TyG quartiles**.

Characteristics		Total (n = 4839)	Quartiles of TyG				*p* value
			Quartile 1	Quartile 2	Quartile 3	Quartile 4	
			(n = 1201)	(n = 1221)	(n = 1214)	(n = 1203)	
			TyG <8.51	8.51 ≤ TyG < 8.92	8.92 ≤ TyG < 9.37	TyG ≥9.37	
Age (years)		65.2 ± 13.8	67.8 ± 14.4	67.0 ± 13.4	64.5 ± 13.6	61.5 ± 12.8	<0.001
Gender, n (%)							0.626
	Male	2993 (61.9)	756 (63.0)	743 (60.9)	741 (61.0)	753 (62.6)	
	Female	1846 (38.1)	445 (37.0)	478 (39.1)	473 (39.0)	450 (37.4)	
Ethnicity, n (%)							0.001
	Caucasian	3668 (75.8)	887 (73.9)	929 (76.1)	934 (76.9)	918 (76.3)	
	African American	663 (13.7)	198 (16.5)	176 (14.4)	155 (12.8)	134 (11.1)	
	Other	508 (10.5)	116 (9.7)	116 (9.5)	125 (10.3)	151 (12.6)	
Vital signs							
	Systolic blood pressure (mmHg)	122.3 ± 18.0	120.7 ± 19.0	121.6 ± 17.2	122.7 ± 17.8	124.5 ± 18.0	<0.001
	Diastolic blood pressure (mmHg)	66.5 ± 10.9	65.8 ± 11.3	66.4 ± 10.8	66.8 ± 10.4	67.2 ± 11.1	0.011
	Mean blood pressure (mmHg)	82.4 ± 12.0	81.2 ± 12.5	82.0 ± 11.8	82.7 ± 11.7	83.5 ± 11.9	<0.001
	Heart rate (beats/min)	85.0 ± 20.6	83.0 ± 20.3	85.0 ± 20.8	85.3 ± 20.4	86.9 ± 20.8	<0.001
	Respiration rate (beats/min)	20.0 ± 5.8	20.0 ± 5.5	20.1 ± 5.7	19.9 ± 6.0	20.1 ± 6.1	0.694
	Oxygen saturation (%)	98 (95, 100)	98 (95, 99)	98 (95, 100)	98 (95, 100)	98 (95, 99)	0.535
	Body mass index (kg/$m^{2}$)	29.5 ± 6.7	27.3 ± 6.5	28.9 ± 6.5	30.0 ± 6.7	31.8 ± 6.4	<0.001
Diagnoses and comorbidities, n (%)							
	Congestive heart failure	793 (16.4)	242 (20.2)	210 (17.2)	180 (14.8)	161 (13.4)	<0.001
	Coronary artery disease	3043 (62.9)	691 (57.5)	776 (63.6)	802 (66.1)	774 (64.3)	<0.001
	Acute coronary syndrome	2295 (47.4)	511 (42.6)	608 (49.8)	601 (49.5)	575 (47.8)	0.001
	STEMI	1035 (21.4)	223 (18.6)	267 (21.9)	275 (22.7)	270 (22.4)	0.050
	NSTEMI	563 (11.6)	122 (10.2)	168 (13.8)	131 (10.8)	142 (11.8)	0.032
	Arrhythmias	1234 (25.5)	358 (29.8)	354 (29.0)	280 (23.1)	242 (20.1)	<0.001
	Cardiac arrest	430 (8.9)	75 (6.2)	98 (8.0)	126 (10.4)	131 (10.9)	<0.001
	Bradycardia	178 (3.7)	59 (4.9)	47 (3.9)	38 (3.1)	34 (2.8)	0.033
	Atrial fibrillation	675 (14.0)	194 (16.2)	199 (16.3)	154 (12.7)	128 (10.6)	<0.001
	Ventricular arrhythmias	344 (7.1)	87 (7.2)	99 (8.1)	82 (6.8)	76 (6.3)	0.355
	Atrioventricular block	127 (2.6)	35 (2.9)	36 (3.0)	30 (2.5)	26 (2.2)	0.569
	Cardiomyopathy	297 (6.1)	85 (7.1)	103 (8.4)	54 (4.5)	55 (4.6)	<0.001
	Valve disease	182 (3.8)	53 (4.4)	54 (4.4)	48 (4.0)	27 (2.2)	0.014
	Shock	975 (20.2)	220 (18.3)	225 (18.4)	251 (20.7)	279 (23.2)	0.008
	Pulmonary embolism	58 (1.2)	15 (1.3)	16 (1.3)	14 (1.2)	13 (1.1)	0.957
	Pulmonary hypertension	49 (1.0)	18 (1.5)	13 (1.1)	11 (0.9)	7 (0.6)	0.156
	Hypertension	1133 (23.4)	291 (24.2)	284 (23.3)	264 (21.8)	294 (24.4)	0.384
	Diabetes	770 (15.9)	97 (8.1)	162 (13.3)	197 (16.2)	314 (26.1)	<0.001
	Hypercholesterolemia	452 (9.3)	94 (7.8)	117 (9.6)	110 (9.1)	131 (10.9)	0.077
	COPD	352 (7.3)	105 (8.7)	98 (8.3)	75 (6.2)	74 (6.2)	0.026
	Respiratory failure	1038 (21.5)	202 (16.8)	248 (20.3)	287 (23.6)	301 (25.0)	<0.001
	Chronic kidney disease	546 (11.3)	149 (12.4)	106 (8.7)	144 (11.9)	147 (12.2)	0.011
	Acute kidney injury	659 (13.6)	128 (10.7)	128 (10.5)	184 (15.2)	219 (18.2)	<0.001
	Malignancy	121 (2.5)	22 (1.8)	35 (2.9)	38 (3.1)	26 (2.2)	0.144
	Stroke	233 (4.8)	65 (5.4)	62 (5.1)	49 (4.0)	57 (4.7)	0.433
	Sepsis	519 (10.7)	105 (8.7)	103 (8.4)	140 (11.5)	171 (14.2)	<0.001
Laboratory parameters							
	White blood cell (${\mathrm{10}}^{9}$/L)	11.3 ± 5.3	10.3 ± 4.8	11.1 ± 5.1	11.9 ± 5.4	12.1 ± 5.8	<0.001
	Lymphocyte percentage (%)	17.8 ± 10.5	17.1 ± 9.8	17.6 ± 10.5	17.5 ± 10.6	18.8 ± 10.9	<0.001
	Monocyte percentage (%)	7.6 ± 2.9	8.0 ± 3.1	7.7 ± 2.8	7.4 ± 2.8	7.3 ± 2.9	<0.001
	Neutrophil percentage (%)	71.9 ± 11.6	72.4 ± 11.4	72.1 ± 11.6	72.3 ± 11.6	70.9 ± 11.8	0.003
	Red blood cell (${\mathrm{10}}^{9}$/L)	4.3 ± 0.8	4.2 ± 0.7	4.2 ± 0.8	4.3 ± 0.8	4.4 ± 0.8	<0.001
	Platelet (${\mathrm{10}}^{9}$/L)	227 ± 83	219 ± 79	227 ± 84	231 ± 81	233 ± 86	<0.001
	Hemoglobin (g/dL)	12.8 ± 2.4	12.6 ± 2.2	12.6 ± 2.5	12.8 ± 2.5	13.2 ± 2.3	<0.001
	Hematocrit (%)	38.5 ± 6.6	38.0 ± 6.1	38.0 ± 6.8	38.5 ± 6.9	39.4 ± 6.5	<0.001
	Glucose (mg/dL)	139.6 ± 39.3	116.6 ± 21.4	129.8 ± 28.1	142.1 ± 34.3	170.1 ± 47.1	<0.001
	Triglyceride (mg/dL)	140.6 ± 109.6	65.3 ± 16.9	98.9 ± 21.4	136.9 ± 33.4	261.7 ± 156.1	<0.001
	Creatinine (mg/dL)	1.44 ± 1.25	1.34 ± 1.12	1.38 ± 1.19	1.49 ± 1.32	1.54 ± 1.37	<0.001
	Blood nitrogen urea (mg/dL)	24.6 ± 17.0	23.3 ± 15.5	24.5 ± 16.7	24.4 ± 16.5	25.9 ± 18.9	0.002
	Sodium (mmol/L)	137.3 ± 4.4	137.3 ± 4.5	137.5 ± 4.3	137.6 ± 4.3	136.9 ± 4.6	<0.001
	Potassium (mmol/L)	4.2 ± 0.7	4.1 ± 0.6	4.1 ± 0.6	4.2 ± 0.7	4.2 ± 0.7	<0.001
	TyG	8.91 ± 0.67	8.26 ± 0.27	8.73 ± 0.12	9.12 ± 0.13	9.73 ± 0.45	<0.001
Medication use, n (%)							
	Antiplatelet	2611 (54.0)	629 (52.4)	662 (54.2)	661 (54.5)	659 (54.8)	0.639
	Oral anticoagulants	375 (7.8)	107 (8.9)	96 (7.9)	94 (7.7)	78 (6.5)	0.173
	Beta-blockers	1877 (38.8)	430 (35.8)	476 (39.0)	481 (39.6)	490 (40.7)	0.079
	ACEI/ARB	1054 (21.8)	270 (22.5)	266 (21.8)	247 (20.4)	271 (22.5)	0.531
	Statin	1680 (34.7)	386 (32.1)	443 (36.3)	421 (34.7)	430 (35.7)	0.145
	APS	35 (25, 50)	33 (24, 46)	35 (25, 48)	36 (25, 53)	38 (26, 56)	<0.001
	APACHE IV	49 (36, 64)	48 (36, 61)	49 (36, 64)	49 (35, 67)	49 (35, 68)	0.146

Continuous variables were presented as mean ± SD or median (IQR). Categorical variables were presented as number (percentage). *p* values were calculated using analysis of variance, Kruskal–Wallis test or Chi-square test to compare differences in variables between different TyG quartiles. Abbreviation: STEMI, ST-elevation myocardial infarction; NSTEMI, non-ST-elevation myocardial infarction; COPD, chronic obstructive pulmonary disease; TyG, triglyceride-glucose index; ACEI, angiotensin-converting enzyme inhibitor; ARB, angiotensin receptor blocker; APS, acute physiology score; APACHE IV, Acute Physiology and Chronic Health Evaluation IV.

### 3.2 Association between PLR and Outcomes

**Fig. 2.**
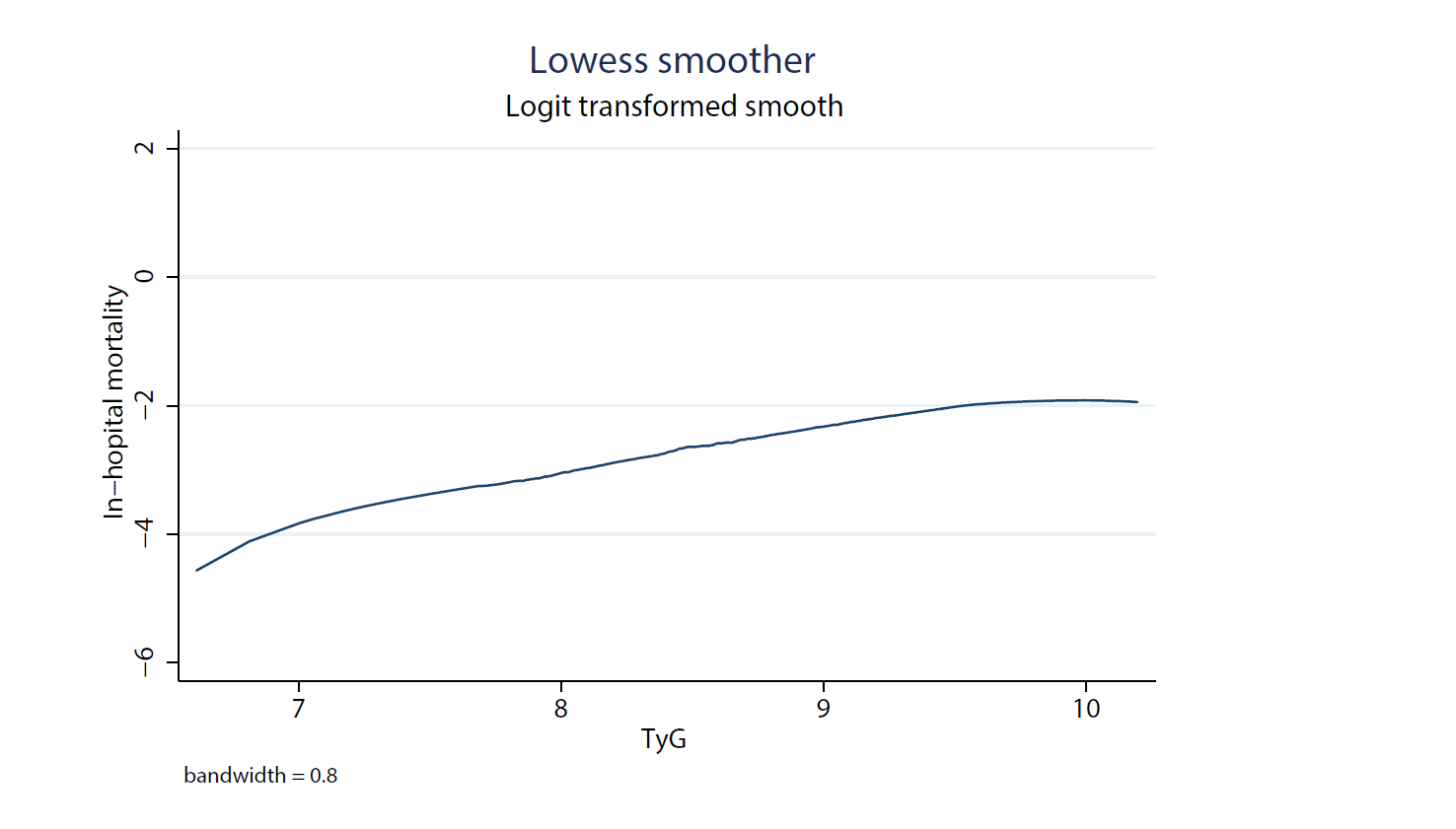
**Local weighted regression was used to plot the curve in line with overall trend, which described the probability of mortality predictded by TyG in raw calculations without adjustment for other covariates**.

Overall, in-hospital mortality rate was 8.5%. As TyG quartiles increased, in-hospital mortality increased significantly (Quartile 4 vs Quartile 1: 12.1 vs 5.3, *p *< 0.001) (Table 2). In unadjusted logistic regression analysis, there was a positive association between TyG and in-hospital mortality (Quartile 4 vs Quartile 1: OR (95% CI): 2.43 (1.79, 3.31), *p *< 0.001, P for trend <0.001). In model 2, after adjusting for age, gender and ethnicity, higher TyG quartiles were markedly associated with the increased risk of mortality (Quartile 4 vs Quartile 1: OR (95% CI): 2.90 (2.12, 3.96), *p *< 0.001, P for trend <0.001). In model 3, adjusted for more confounding variables, the TyG index was still independently related to the increased risk of in-hospital mortality (Quartile 4 vs Quartile 1: OR (95% CI): 1.83 (1.27, 2.64), *p *< 0.001, P for trend <0.001). Furthermore, when TyG was considered as a continuous variable in the model for analysis, we observed that for each unit increase in the TyG index, the risk of in-hospital mortality increased approximately 0.35-fold in Model 1 (*p *< 0.001), 0.43-fold in Model 2 (*p *< 0.001), 0.23-fold in Model 3 (*p *< 0.001) respectively (Table 3). Interestingly, of the 4069 patients who didn’t suffer from diabetes, we found that TyG had a significant effect on in-hospital mortality with or without adjusting for confounding variables, which was consistent with the conclusion drawn in Table 3. Conversely, as we screened patients with diabetes (N = 770) for logistics regression analysis, no significant correlation has been shown between TyG and in-hospital mortality with or without adjusting for confounding risk factors (Table 4). Besides, from Lowess curve in Fig. 2, we found that the relationship between TyG and mortality was linear, as TyG increased, in-hospital mortality increased.

**Table 2.** **Outcomes of patients stratified by TyG quartiles**.

Outcomes	Total (n = 4839)	Quartiles of TyG				*p* value
		Quartile 1	Quartile 2	Quartile 3	Quartile 4	
		(n = 1201)	(n = 1221)	(n = 1214)	(n = 1203)	
		TyG <8.51	8.51 ≤ TyG < 8.92	8.92 ≤ TyG < 9.37	TyG ≥9.37	
In-hospital mortality, n (%)	413 (8.5)	64 (5.3)	87 (7.1)	117 (9.6)	145 (12.1)	<0.001
Length of ICU stay (days)	2.2 (1.3 4.3)	2.1 (1.3, 3.8)	2.2 (1.3, 4.2)	2.4 (1.3, 4.7)	2.3 (1.3, 4.9)	0.007
Length of hospital stay (days)	5.9 (3.3, 11.1)	5.7 (3.2, 9.8)	6.0 (3.5, 11.5)	6.2 (3.3, 11.7)	5.9 (3.1, 11.5)	0.100

Continuous variables were presented as median (IQR). Categorical variables were presented as number (percentage). *p* values were calculated using Kruskal–Wallis test or Chi-square test to compare differences in outcomes between different TyG quartiles. Abbreviation: TyG, triglyceride-glucose index; ICU, intensive care unit.

**Table 3.** **The association between TyG and in-hospital mortality**.

		OR (95% CI)	*p* value	P for trend
Model 1				<0.001
	Quartile 1: TyG <8.51	Reference		
	Quartile 2: 8.51 ≤ TyG < 8.92	1.36 (0.98, 1.90)	0.068	
	Quartile 3: 8.92 ≤ TyG < 9.37	1.89 (1.38, 2.60)	<0.001	
	Quartile 4: TyG ≥9.37	2.43 (1.79, 3.31)	<0.001	
	Continuous	1.35 (1.23, 1.48)	<0.001	
Model 2				<0.001
	Quartile 1: TyG <8.51	Reference		
	Quartile 2: 8.51 ≤ TyG < 8.92	1.40 (1.00, 1.95)	0.051	
	Quartile 3: 8.92 ≤ TyG < 9.37	2.07 (1.50, 2.85)	<0.001	
	Quartile 4: TyG ≥9.37	2.90 (2.12, 3.96)	<0.001	
	Continuous	1.43 (1.30, 1.58)	<0.001	
Model 3				<0.001
	Quartile 1: TyG <8.51	Reference		
	Quartile 2: 8.51 ≤ TyG < 8.92	1.15 (0.80, 1.68)	0.448	
	Quartile 3: 8.92 ≤ TyG < 9.37	1.47 (1.03, 2.11)	0.035	
	Quartile 4: TyG ≥9.37	1.83 (1.27, 2.64)	0.001	
	Continuous	1.23 (1.10, 1.38)	<0.001	

Models were derived from binary logistic regression analysis. P for trend was calculated using binary logistic analysis to determine whether there was a trend when TyG was included as a grouping variable in the model (Quartile 1–4). When TyG was included as a grouping variable in the model, *p* values were calculated using binary logistic analysis to determine whether there was a relationship between TyG quartiles and in-hospital mortality with Quartile1 serving as the reference group. When TyG was included as a continuous variable in the model, *p* values were calculated using binary logistic analysis to determine whether there was a relationship between TyG and in-hospital mortality. Model 1: unadjusted. Model 2: adjusted for age, gender, ethnicity. Model 3: adjusted for age, gender, ethnicity, systolic blood pressure, diastolic blood pressure, respiration, congestive heart failure, STEMI, cardiac arrest, acute kidney injury, respiratory failure, stroke, malignancy, white blood cell, neutrophil percentage, oral anticoagulants, ACEI/ARB, APACHE IV, length of ICU stay and length of hospital stay. Abbreviation: TyG, triglyceride-glucose index; STEMI, ST-elevation myocardial infarction; ACEI, angiotensin-converting enzyme inhibitor; ARB, angiotensin receptor blocker; APACHE IV, Acute Physiology and Chronic Health Evaluation IV; ICU, intensive care unit; OR, odds ratio; CI, confidence interval.

**Table 4.** **The association between TyG and in-hospital mortality in patients with DM or no-DM**.

DM		OR (95% CI)	*p* value	P for trend	No-DM		OR (95% CI)	*p* value	P for trend
Model 1				0.291	Model 1				<0.001
	Quartile 1: TyG <8.51	Reference				Quartile 1: TyG <8.51	Reference		
	Quartile 2: 8.51 ≤ TyG < 8.92	1.14 (0.46, 2.79)	0.782			Quartile 2: 8.51≤ TyG <8.92	1.37 (0.95, 1.96)	0.090	
	Quartile 3: 8.92 ≤ TyG < 9.37	1.33 (0.57, 3.12)	0.515			Quartile 3: 8.92 ≤ TyG < 9.37	1.95 (1.39, 2.74)	<0.001	
	Quartile 4: TyG ≥9.37	1.44 (0.65, 3.21)	0.373			Quartile 4: TyG ≥9.37	2.62 (1.87, 3.66)	<0.001	
	Continuous	1.13 (0.90, 1.41)	0.297			Continuous	1.38 (1.25, 1.53)	<0.001	
Model 2				0.009	Model 2				<0.001
	Quartile 1: TyG <8.51	Reference				Quartile 1: TyG <8.51	Reference		
	Quartile 2: 8.51 ≤ TyG < 8.92	1.14 (0.46, 2.81)	0.781			Quartile 2: 8.51 ≤ TyG < 8.92	1.41 (0.98, 2.02)	0.062	
	Quartile 3: 8.92 ≤ TyG < 9.37	1.37 (0.58, 3.24)	0.477			Quartile 3: 8.92 ≤ TyG < 9.37	2.15 (1.52, 3.03)	<0.001	
	Quartile 4: TyG ≥9.37	1.58 (0.69, 3.60)	0.280			Quartile 4: TyG ≥9.37	3.16 (2.24, 4.46)	<0.001	
	Continuous	1.17 (0.92, 1.48)	0.196			Continuous	1.48 (1.33, 1.64)	<0.001	
Model 3				<0.001	Model 3				<0.001
	Quartile 1: TyG <8.51	Reference				Quartile 1: TyG <8.51	Reference		
	Quartile 2: 8.51 ≤ TyG < 8.92	1.38 (0.47, 4.04)	0.554			Quartile 2: 8.51 ≤ TyG < 8.92	1.25 (0.83, 1.89)	0.283	
	Quartile 3: 8.92 ≤ TyG < 9.37	1.79 (0.63, 5.04)	0.273			Quartile 3: 8.92 ≤ TyG < 9.37	1.57 (1.05, 2.36)	0.028	
	Quartile 4: TyG ≥9.37	2.37 (0.87, 6.46)	0.091			Quartile 4: TyG ≥9.37	2.08 (1.38, 3.16)	0.001	
	Continuous	1.32 (0.99, 1.77)	0.057			Continuous	1.28 (1.12, 1.46)	<0.001	

Models were derived from binary logistic regression analysis. P for trend was calculated using binary logistic analysis to determine whether there was a trend when TyG was included as a grouping variable in the model (Quartile 1–4). When TyG was included as a grouping variable in the model, *p* values were calculated using binary logistic analysis to determine whether there was a relationship between TyG quartiles and in-hospital mortality with Quartile1 serving as the reference group. When TyG was included as a continuous variable in the model, *p* values were calculated using binary logistic analysis to determine whether there was a relationship between TyG and in-hospital mortality. In DM group: Model 1: unadjusted. Model 2: adjusted for age, gender, ethnicity. Model 3: adjusted for age, gender, ethnicity, systolic blood pressure, respiratory failure, stroke, pulmonary embolism, hemoglobin, hematocrit, APACHE IV. In No-DM group: Model 1: unadjusted. Model 2: adjusted for age, gender, ethnicity. Model 3: adjusted for age, gender, ethnicity, systolic blood pressure, diastolic blood pressure, congestive heart failure, STEMI, cardiac arrest, malignancy, respiratory failure, shock, stroke, acute kidney injury, white blood cell, neutrophil percentage, sodium, oral anticoagulants, ACEI/ARB, APS, APACHE IV, length of ICU stay and length of hospital stay. Abbreviation: TyG, triglyceride-glucose index; DM, diabetes; STEMI, ST-elevation myocardial infarction; ACEI, angiotensin-converting enzyme inhibitor; ARB, angiotensin receptor blocker; APS, acute physiology score; APACHE IV, Acute Physiology and Chronic Health Evaluation IV; ICU, intensive care unit; OR, odds ratio; CI, confidence interval.

**Fig. 3.**
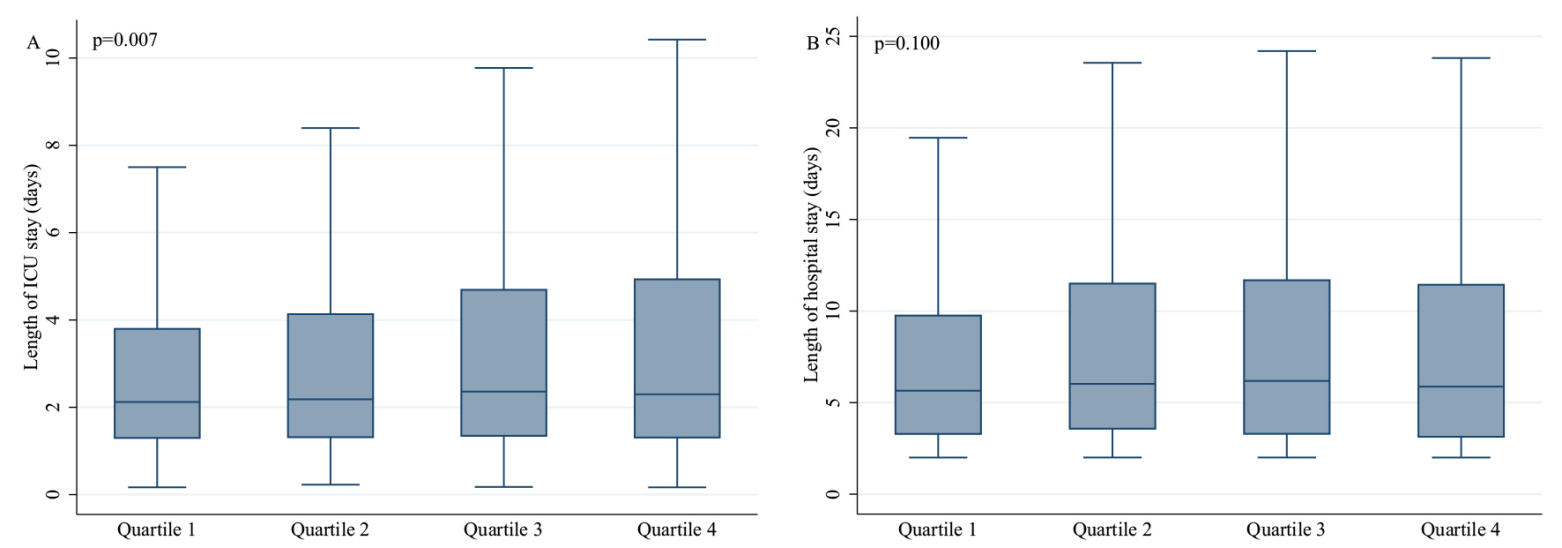
**Association between the triglyceride-glucose index and the length of ICU and hospital stay through box plot**. Abbreviation: ICU, intensive care unit.

Besides, increased TyG quartiles were associated with prolonged length of ICU stay (Quartile 4 vs Quartile 1: 2.3 (1.3, 4.9) vs 2.1 (1.3, 3.8), *p* = 0.007), while with the growth of the TyG index, the length of hospital stay failed to increase significantly (Quartile 4 vs Quartile 1: 5.9 (3.1, 11.5) vs 5.7 (3.2, 9.8), *p* = 0.100) (Table 2). Moreover, we drew the box plot to reflect the relationship between TyG and length of ICU and hospital stay more intuitively. The obvious association between TyG and length of ICU stay was indicated (Fig. 3).

**Fig. 4.**
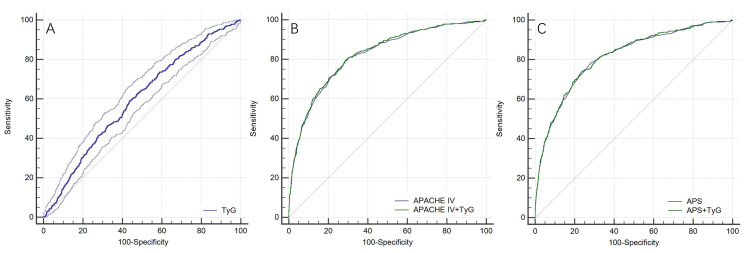
** ROC curves for the prediction of in-hospital mortality**. (A) ROC curve for the prediction of in-hospital mortality of Tyg. (B) ROC curves for the prediction of in-hospital mortality of APACHE IV and APACHE IV+TyG. (C) ROC curves for the prediction of in-hospital mortality of APS and APS+TyG. Abbreviation: ROC, receiver-operating characteristic; TyG, triglyceride-glucose index; APACHE IV, Acute Physiology and Chronic Health Evaluation IV; APS, acute physiology score.

The ROC curve revealed a moderate ability of TyG to predict in-hospital mortality (AUC = 0.594 (0.580, 0.608)), the optimal cutoff value was 8.99, the sensitivity was 59.08%, and the specificity was 56.17%. The AUC of APACHE IV was 0.821 (0.810, 0.832), when combined with TyG, the AUC increased to 0.824 (0.813, 0.835), but there was no statistically significant difference (*p* = 0.069). The AUC of APS was 0.813 (0.802, 0.824), when combined with TyG, the AUC increased to 0.815 (0.804, 0.826), there was still no statistically significant difference (*p* = 0.254) (Fig. 4).

### 3.3 Subgroup Analysis

Patients complicated by arrhythmias or atrial fibrillation had higher risk of in-hospital mortality for TyG while patients with sepsis had lower risk of in-hospital mortality for TyG (Table 5).

**Table 5.** **Subgroup analysis of associations between in-hospital mortality and TyG**.

Subgroups		N	Quartile 1	Quartile 2	Quartile 3	Quartile 4	P for interaction
Age (years)							0.259
	<66	2376	Reference	1.44 (0.80, 2.60)	1.70 (0.97, 2.98)	2.40 (1.42, 4.05)	
	≥66	2463	Reference	1.35 (0.90, 2.02)	2.20 (1.50, 3.24)	3.12 (2.12, 4.60)	
Gender							0.659
	Male	2993	Reference	1.52 (0.99, 2.34)	1.84 (1.21, 2.79)	2.44 (1.63, 3.64)	
	Female	1846	Reference	1.15 (0.68, 1.94)	1.95 (1.21, 3.17)	2.43 (1.51, 3.90)	
Ethnicity							0.722
	Caucasian	3668	Reference	1.45 (0.99, 2.11)	1.89 (1.31, 2.71)	2.38 (1.67, 3.39)	
	African American	663	Reference	1.58 (0.62, 4.03)	3.52 (1.51, 8.22)	5.45 (2.37, 12.50)	
	Other	508	Reference	0.61 (0.19, 1.92)	0.68 (0.23, 2.02)	0.96 (0.37, 2.51)	
Body mass index (kg/$m^{2}$)							0.707
	<29.5	2679	Reference	1.56 (1.03, 2.35)	2.47 (1.67, 3.65)	2.62 (1.74, 3.96)	
	≥29.5	2160	Reference	1.07 (0.60, 1.89)	1.27 (0.74, 2.18)	2.15 (1.31, 3.52)	
Systolic blood pressure (mmHg)							0.239
	<122	2592	Reference	1.49 (1.00, 2.23)	1.85 (1.25, 2.73)	2.66 (1.82, 3.90)	
	≥122	2247	Reference	1.17 (0.64, 2.13)	2.09 (1.21, 3.59)	2.46 (1.46, 4.14)	
Diastolic blood pressure (mmHg)							0.159
	<67	2647	Reference	1.37 (0.93, 2.00)	1.72 (1.19, 2.49)	2.62 (1.84, 3.72)	
	≥67	2790	Reference	1.52 (0.75, 3.07)	2.72 (1.42, 5.18)	2.48 (1.30, 4.76)	
Mean blood pressure (mmHg)							0.078
	<82	2552	Reference	1.36 (0.91, 2.04)	1.92 (1.31, 2.83)	2.86 (1.97, 4.15)	
	≥82	2287	Reference	1.44 (0.80, 2.60)	1.99 (1.34, 3.48)	2.17 (1.25, 3.74)	
Heart rate (beats/min)							0.449
	<97	1781	Reference	1.35 (0.81, 2.25)	1.85 (1.14, 3.00)	2.43 (1.52, 3.88)	
	≥85	2226	Reference	1.51 (0.98, 2.35)	1.88 (1.23, 2.87)	2.37 (1.57, 3.57)	
Respiration rate (beats/min)							0.063
	<20	2444	Reference	1.27 (0.75, 2.15)	1.54 (0.93, 2.55)	1.94 (1.18, 3.17)	
	≥20	2395	Reference	1.43 (0.93, 2.20)	2.21 (1.47, 3.33)	2.81 (1.90, 4.17)	
Oxygen saturation (%)							0.695
	<97	2814	Reference	1.21 (0.80, 1.81)	1.71 (1.18, 2.48)	2.41 (1.69, 3.44)	
	≥97	3058	Reference	1.39 (0.89, 2.15)	1.94 (1.28, 2.94)	2.45 (1.64, 3.68)	
Congestive heart failure							0.150
	Yes	793	Reference	2.05 (1.05, 3.99)	2.91 (1.51, 5.60)	4.05 (2.13, 7.72)	
	No	4046	Reference	1.21 (0.83, 1.78)	1.73 (1.20, 2.48)	2.21 (1.56, 3.14)	
Coronary artery disease							0.095
	Yes	3043	Reference	1.10 (0.67, 1.83)	1.42 (0.88, 2.28)	1.92 (1.22, 3.03)	
	No	1796	Reference	1.76 (1.12, 2.76)	2.78 (1.81, 4.27)	3.35 (2.21, 5.09)	
Acute coronary syndrome							0.940
	Yes	2295	Reference	1.02 (0.55, 1.89)	1.55 (0.87, 2.76)	2.31 (1.33, 3.98)	
	No	2544	Reference	1.67 (1.12, 2.49)	2.24 (1.53, 3.28)	2.65 (1.83, 3.84)	
STEMI							0.223
	Yes	1035	Reference	1.17 (0.37, 3.75)	1.65 (0.55, 4.89)	3.68 (1.36, 9.92)	
	No	3804	Reference	1.43 (1.01, 2.02)	2.00 (1.44, 2.79)	2.39 (1.73, 3.30)	
NSTEMI							0.755
	Yes	563	Reference	1.80 (0.62, 5.25)	1.52 (0.48, 4.79)	2.56 (0.89, 7.32)	
	No	4276	Reference	1.33 (0.93, 1.89)	1.93 (1.39, 2.69)	2.44 (1.77, 3.35)	
Arrhythmias							0.006
	Yes	1234	Reference	1.37 (0.73, 2.58)	3.33 (1.87, 5.93)	4.20 (2.36, 7.46)	
	No	3605	Reference	1.36 (0.92, 2.01)	1.51 (1.04, 2.21)	2.03 (1.42, 2.92)	
Cardiac arrest							0.694
	Yes	430	Reference	0.90 (0.45, 1.82)	1.32 (0.69, 2.51)	2.13 (1.14, 3.99)	
	No	4409	Reference	1.45 (0.98, 2.15)	1.86 (1.27, 2.70)	2.20 (1.52, 3.18)	
Bradycardia							0.444
	Yes	178	Reference	0.94 (0.20, 4.41)	1.18 (0.25, 5.58)	1.33 (0.28, 6.33)	
	No	4661	Reference	1.39 (0.99, 1.96)	1.94 (1.40, 2.68)	2.49 (1.82, 3.41)	
Atrial fibrillation							0.018
	Yes	675	Reference	1.76 (0.81, 3.79)	3.54 (1.69, 7.39)	5.09 (2.45, 10.60)	
	No	4164	Reference	1.28 (0.89, 1.86)	1.67 (1.18, 2.37)	2.16 (1.54, 3.02)	
Ventricular arrhythmias							0.111
	Yes	237	Reference	0.69 (0.26, 1.82)	1.22 (0.50, 2.96)	2.06 (0.86, 4.92)	
	No	4495	Reference	1.35 (0.96, 1.92)	1.83 (1.31, 2.54)	2.29 (1.66, 3.16)	
Atrioventricular block							0.821
	Yes	127	Reference	0.47 (0.04, 5.45)	1.83 (0.29, 11.78)	2.15 (0.33, 13.92)	
	No	4712	Reference	1.39 (0.99, 1.95)	1.90 (1.38, 2.61)	2.44 (1.79, 3.33)	
Cardiomyopathy							0.554
	Yes	297	Reference	1.48 (0.42, 5.22)	2.07 (0.53, 8.07)	3.45 (0.98, 12.07)	
	No	4542	Reference	1.36 (0.96, 1.92)	1.88 (1.36, 2.60)	2.38 (1.74, 3.27)	
Valve disease							0.952
	Yes	182	Reference	3.33 (0.85, 13.08)	1.94 (0.44, 8.58)	3.79 (0.83, 17.26)	
	No	4657	Reference	1.28 (0.90, 1.80)	1.89 (1.37, 2.62)	2.41 (1.76, 3.29)	
Shock							0.067
	Yes	975	Reference	1.08 (0.65, 1.79)	1.36 (0.84, 2.20)	1.63 (1.03, 2.58)	
	No	3864	Reference	1.68 (1.06, 2.66)	2.37 (1.53, 3.68)	3.05 (1.98, 4.68)	
Pulmonary embolism							0.006
	Yes	43	,	0.89 (0.01, 0.57)	0.47 (0.10, 2.18)	,	
	No	4781	Reference	1.33 (0.95, 1.86)	1.79 (1.30, 2.46)	2.28 (1.68, 3.10)	
Pulmonary hypertension							0.249
	Yes	49	Reference	0.91 (0.13, 6.40)	0.50 (0.05, 5.51)	0.83 (0.07, 9.69)	
	No	4790	Reference	1.39 (0.99, 1.95)	1.96 (1.42, 2.71)	2.52 (1.84, 3.44)	
Hypertension							0.814
	Yes	1133	Reference	1.28 (0.60, 2.70)	1.75 (0.85, 3.60)	2.25 (1.14, 4.44)	
	No	3706	Reference	1.38 (0.95, 2.00)	1.92 (1.35, 2.72)	2.49 (1.77, 3.51)	
Diabetes							0.109
	Yes	770	Reference	1.14 (0.46, 2.79)	1.33 (0.57, 3.12)	1.44 (0.65, 3.21)	
	No	4069	Reference	1.37 (0.95, 1.96)	1.95 (1.39, 2.74)	2.62 (1.87, 3.66)	
Hypercholesterolemia							0.199
	Yes	452	Reference	1.65 (0.48, 5.66)	1.53 (0.43, 5.39)	1.46 (0.43, 5.01)	
	No	4387	Reference	1.34 (0.95, 1.90)	1.93 (1.39, 2.68)	2.56 (1.86, 3.51)	
COPD							0.833
	Yes	352	Reference	1.35 (0.53, 3.41)	1.83 (0.72, 4.67)	2.71 (1.12, 6.59)	
	No	4487	Reference	1.37 (0.96, 1.96)	1.94 (1.39, 2.72)	2.46 (1.78, 3.42)	
Respiratory failure							0.419
	Yes	1038	Reference	1.27 (0.77, 2.11)	1.58 (0.98, 2.55)	2.41 (1.52, 3.81)	
	No	3801	Reference	1.28 (0.81, 2.03)	1.79 (1.16, 2.77)	1.88 (1.21, 2.91)	
Chronic kidney disease							0.388
	Yes	546	Reference	0.54 (0.20, 1.43)	1.79 (0.90, 3.56)	2.49 (1.28, 4.82)	
	No	4293	Reference	1.60 (1.11, 2.31)	1.95 (1.36, 2.78)	2.45 (1.73, 3.47)	
Acute kidney injury							0.574
	Yes	659	Reference	1.29 (0.69, 2.41)	1.80 (1.02, 3.16)	1.86 (1.08, 3.21)	
	No	4180	Reference	1.43 (0.95, 2.13)	1.71 (1.15, 2.54)	2.29 (1.57, 3.35)	
Malignancy							0.426
	Yes	121	Reference	0.70 (0.19, 2.66)	1.06 (0.30, 3.67)	1.25 (0.33, 4.69)	
	No	4718	Reference	1.39 (0.98, 1.97)	1.92 (1.38, 2.67)	2.52 (1.84, 3.46)	
Sepsis							<0.001
	Yes	519	Reference	1.27 (0.67, 2.43)	1.07 (0.58, 1.99)	0.94 (0.51, 1.71)	
	No	4320	Reference	1.45 (0.97, 2.17)	2.18 (1.50, 3.19)	3.02 (2.10, 4.36)	
Stroke							0.754
	Yes	233	Reference	1.25 (0.40, 3.96)	2.52 (0.85, 7.50)	2.62 (0.91, 7.52)	
	No	4606	Reference	1.38 (0.97, 1.95)	1.88 (1.35, 2.62)	2.44 (1.77, 3.36)	
Antiplatelet							0.793
	Yes	2611	Reference	2.01 (1.18, 3.43)	2.32 (1.37, 3.91)	2.79 (1.67, 4.66)	
	No	2228	Reference	1.05 (0.68, 1.63)	1.72 (1.15, 2.58)	2.34 (1.59, 3.45)	
Oral anticoagulants							0.765
	Yes	375	Reference	2.88 (0.55, 15.23)	4.22 (0.86, 20.86)	2.10 (0.34, 12.88)	
	No	4464	Reference	1.31 (0.93, 1.84)	1.81 (1.31, 2.50)	2.40 (1.76, 3.28)	
Beta, blockers							0.242
	Yes	1877	Reference	2.51 (1.28, 4.94)	2.23 (1.13, 4.44)	4.22 (2.23, 8.02)	
	No	2962	Reference	1.10 (0.74, 1.63)	1.89 (1.32, 2.70)	2.05 (1.43, 2.93)	
ACEI/ARB							0.221
	Yes	1054	Reference	3.42 (1.10, 10.62)	1.94 (0.56, 6.71)	2.28 (0.69, 7.51)	
	No	3785	Reference	1.22 (0.86, 1.74)	1.86 (1.34, 2.59)	2.48 (1.80, 3.41)	
Statin							0.348
	Yes	1680	Reference	2.23 (0.97, 5.13)	3.37 (1.52, 7.48)	3.93 (1.79, 8.61)	
	No	3159	Reference	1.28 (0.88, 1.85)	1.71 (1.21, 2.43)	2.30 (1.64, 3.22)	
White blood cell (${\mathrm{10}}^{9}$/L)							0.763
	<11.3	2849	Reference	1.63 (1.00, 2.66)	1.60 (0.97, 2.65)	2.21 (1.37, 3.57)	
	≥11.3	1990	Reference	1.06 (0.67, 1.67)	1.72 (1.13, 2.60)	2.13 (1.42, 3.21)	
Lymphocyte percentage (%)							0.428
	<17.8	3159	Reference	1.60 (1.09, 2.33)	2.18 (1.52, 3.13)	2.68 (1.87, 3.84)	
	≥17.8	1680	Reference	0.80 (0.38, 1.66)	1.30 (0.68, 2.50)	2.15 (1.18, 3.89)	
Monocyte percentage (%)							0.542
	<7.6	2972	Reference	1.20 (0.79, 1.83)	1.74 (1.17, 2.58)	2.43 (1.67, 3.53)	
	≥7.6	1876	Reference	1.65 (0.96, 2.84)	2.12 (1.25, 3.61)	2.22 (1.30, 3.79)	
Neutrophil percentage (%)							0.122
	<71.9	1548	Reference	0.74 (0.33, 1.64)	0.91 (0.42, 1.96)	2.06 (1.08, 3.93)	
	≥71.9	3291	Reference	1.57 (1.09, 2.28)	2.22 (1.56, 3.15)	2.66 (1.87, 3.76)	
Red blood cell (${\mathrm{10}}^{9}$/L)							0.320
	<4.3	2270	Reference	1.38 (0.90, 2.12)	2.20 (1.47, 3.28)	3.17 (2.14, 4.69)	
	≥4.3	2569	Reference	1.40 (0.82, 2.41)	1.69 (1.01, 2.83)	2.06 (1.25, 3.38)	
Platelet (${\mathrm{10}}^{9}$/L)							0.683
	<227	2688	Reference	1.39 (0.91, 2.12)	1.84 (1.22, 2.78)	2.42 (1.63, 3.60)	
	≥227	2151	Reference	1.34 (0.79, 2.29)	1.99 (1.21, 3.27)	2.49 (1.54, 4.04)	
Hemoglobin (g/dL)							0.691
	<12.8	2175	Reference	1.41 (0.94, 2.13)	1.82 (1.22, 2.71)	2.83 (1.91, 4.18)	
	≥12.8	2664	Reference	1.33 (0.75, 2.36)	2.20 (1.30, 3.71)	2.55 (1.53, 4.23)	
Hematocrit (%)							0.810
	<38.5	2201	Reference	1.37 (0.89, 2.11)	1.94 (1.28, 2.94)	2.82 (1.88, 4.24)	
	≥38.5	2638	Reference	1.39 (0.82, 2.34)	1.93 (1.18, 3.16)	2.36 (1.47, 3.78)	
Creatinine (mg/dL)							0.670
	<1.44	3525	Reference	1.22 (0.78, 1.89)	1.68 (1.10, 2.56)	2.15 (1.43, 3.23)	
	≥1.44	1314	Reference	1.57 (0.93, 2.64)	1.96 (1.20, 3.20)	2.44 (1.51, 3.92)	
Blood nitrogen urea (mg/dL)							0.356
	<24.6	3236	Reference	1.45 (0.91, 2.33)	1.93 (1.23, 3.02)	2.46 (1.59, 3.80)	
	≥24.6	1603	Reference	1.24 (0.77, 2.01)	1.84 (1.17, 2.89)	2.29 (1.49, 3.54)	
Sodium (mmol/L)							0.722
	<137	1802	Reference	1.72 (1.02, 2.90)	2.26 (1.37, 3.75)	2.12 (1.30, 3.48)	
	≥137	3037	Reference	1.15 (0.74, 1.78)	1.68 (1.12, 2.52)	2.66 (1.80, 3.92)	
Potassium (mmol/L)							0.255
	<4.2	2638	Reference	0.99 (0.63, 1.58)	1.25 (0.81, 1.95)	1.94 (1.28, 2.95)	
	≥4.2	2201	Reference	1.93 (1.18, 3.17)	2.91 (1.82, 4.65)	3.09 (1.95, 4.88)	
APS							0.086
	<41	2937	Reference	1.77 (0.86, 3.65)	2.03 (0.99, 4.16)	1.95 (0.94, 4.05)	
	≥41	1902	Reference	1.14 (0.77, 1.69)	1.53 (1.06, 2.21)	2.01 (1.41, 2.86)	
APACHE IV							0.155
	<53	2799	Reference	1.92 (0.91, 4.03)	1.65 (0.77, 3.56)	2.08 (0.99, 4.34)	
	≥53	2040	Reference	1.17 (0.80, 1.72)	1.86 (1.30, 2.66)	2.46 (1.74, 3.49)	

Binary logistic regression analysis was used and results were presented as OR (odds ratio) and 95% CI (confidence interval). P for interaction was calculated using binary logistic analysis to determine whether there is interaction between different subgroups and TyG quartiles. Abbreviation: STEMI, ST-elevation myocardial infarction; NSTEMI, non-ST-elevation myocardial infarction; COPD, chronic obstructive pulmonary disease; triglyceride-glucose index; ACEI, angiotensin-converting enzyme inhibitor; ARB, angiotensin receptor blocker; APS, acute physiology score; APACHE IV, Acute Physiology and Chronic Health Evaluation IV.

## 4. Discussion

This study affirmed the relationship between TyG and in-hospital mortality in critically ill patients with heart disease. The highlights of this study are as follows: (1) TyG index was a strong indicator of in-hospital mortality in critically ill patients with heart disease, even after adjusting for possible confounding variables. Whereas, we failed to reveal a significant association between the TyG index and in-hospital mortality in patients with diabetes. (2) The Lowess curve presented a positive relationship between TyG and in-hospital mortality. (3) Significant interactions were not observed in most subgroups. (4) Length of ICU was prolonged as TyG increased.

Previous studies have indicated that IR was strongly associated with the development and prognosis of CVD [22, 23, 24]. As an alternative method for evaluating, IR is a well-recognized risk factor for cardiovascular disease that induces an imbalance in glucose metabolism, leading to hyperglycemia, triggering inflammation and oxidative stress, systemic lipid disorders, which may contribute to the development of atherosclerosis [25]. In addition, studies have shown that IR can induce an increase in glycosylation products and free radicals, leading to inactivation of nitric oxide (NO), activation of the mitochondrial electron transport chain, and overproduction of reactive oxidative stress (ROS), which damage blood vessels endothelium [26, 27]. Moreover, IR can increase the expression of adhesion-inducing and thromboxane A2 (TxA2)-dependent tissue factor in platelets. These are associated with thrombosis and inflammation [28]. Furthermore, IR can induce excessive glycosylation, promote smooth muscle cell proliferation, collagen cross-linking, and collagen deposition, leading to increased left ventricular stiffness, cardiac fibrosis, and ultimately heart failure [29]. In addition, IR-induced activation of the renin-angiotensin system [30] and impaired cardiac calcium processing [31] may also contribute to the development of cardiovascular disease. As we know, the euglycemic-hyperinsulinemic clamp is the gold standard method for the diagnosis of IR [32]. However, due to the high cost and complex operation of this method, it is relatively difficult to carry out in practical clinical application. The homeostasis model assessment of insulin resistance (HOMA-IR) is a substitutive method for IR evaluation [33]. While it requires insulin concentration which is not routine clinical examination item. In this respect, TyG index which is calculated by fasting TGs and glucose is more readily available in clinical practice. And it has been proven to have a good predictive ability on IR compared with the above-mentioned two methods [34, 35]. Therefore, as a good substitute indicator for IR, TyG index may be a risk factor which associated with prognosis of CVD.

TyG has been extensively demonstrated to be significantly related to the development of a variety of diseases in former studies. A recent meta-analysis which included 13 cohort studies confirmed that TyG index was strongly related to the incidence of diabetes [36]. Furthermore, higher TyG index has been indicated to be associated with the increased risk of ischemic stroke [37]. Similarly, a large number of studies have also confirmed the relationship between TyG and CVD. A previous prospective cohort study proved that higher TyG index was related to the increased complexity of coronary lesions and the risk of worse outcomes in patients with NSTE-ACS [38]. Zhao *et al*. [16] enrolled 798 patients with T2DM and NSTEACS who underwent PCI and revealed that the level of TyG index was strongly associated with the incidence of adverse cardiovascular event during a 36-month follow-up. Luo *et al*. [38] reached the same conclusion in STEMI patients who were treated with PCI. Besides, TyG index was also proved to be an independent predictor of major cardiovascular events in patients with T2DM complicated by ACS undergoing PCI [39]. Even among patients with stable CAD, higher TyG index was still associated with the increased risk of mortality [40, 41]. Thus, paying attention to TyG in clinical practice and improving the level of nursing, monitoring may improve the prognosis and reduce mortality.

This study drawn a similar conclusion that increased TyG was independently related to the in-hospital mortality in critically ill patients with heart disease, providing evidence for the use of TyG in patients with severe CVD. While, when conducting multiple logistic regression analysis, there was no significant association between TyG and in-hospital mortality among patients with diabetes in model 1–3. The discrepancy might be explained by the small number of patients with diabetes in the cohort.

Interestingly, gender differences appeared to have an impact on the prediction of adverse outcomes of TyG. The previous study has shown that the ability of TyG to predict adverse cardiovascular events was better in women than men when TyG >9.53 [19]. The plausible explanation might be that female patients with diabetes had a higher incidence of CVD, especially in post-menopausal women [42]. Moreover, the role of hormones cannot be ignored. However, in the gender subgroup in our study, we failed to find the obvious interaction (*p* = 0.659). The reason might be that patients enrolled in this study have clearly been diagnosed with CVD and mortality of those was extremely high. Therefore, sex differences were attenuated.

Through the Lowess curve, we found that in-hospital mortality increased as the increase of TyG value. This was consistent with the conclusion that TyG was an independent predictor when considered as a continuous variable in multivariate logistic regression, which reconfirmed the reliability of TyG application in critically ill patients with heart disease.

In addition, higher TyG quartiles were associated with the increased length of ICU stay, which brought the psychological, physical, and financial burden on patients. Most of critically ill patients with heart disease have limited mobility so that complex clinical examination cannot be performed. In this circumstance, some of complex predictive scores can’t be calculated. Therefore, easily accessible indicators like TyG are more cost-effective and important for ICU patients.

## 5. Limitation

This study is a single-center retrospective cohort study. Due to the limitations of the retrospective study, selection bias and recall bias cannot be avoided, and the causal relationship cannot be determined. Moreover, the severity for each kind of heart disease can not be stratified and the cause-of-death data was unavailable due to the limitation of our database. Furthermore, in patients with diabetes, the accuracy of the model is reduced because of the small sample size. And we are not able to demonstrate whether the appropriate treatment which aimed to reduce the TyG value related to the lower incidence of adverse clinical outcomes.

## 6. Conclusions

To summarize, the results indicated that TyG was an independent predictor of in-hospital mortality in critically ill patients with heart disease. And through multivariate logistic regression, the in-hospital mortality increased significantly as TyG quartiles increased. When considered as a continuous variable, TyG has been proven to significantly related to adverse events. In subgroup analysis, no significant interactions were observed in most subgroups. Furthermore, high TyG was associated with prolonged ICU stay length.

## Data Availability

The data used in this study was from eICU Collaborative Research Database [24], which is available at: https://doi.org/10.13026/C2WM1R. The author was approved to access to the database through Protecting Human Research Participants exam (certificate number: 9728458).

## Method Statement

All methods were carried out in accordance with relevant guidelines and regulations.

## Supplementary Material

Supplementary material associated with this article can be found, in the online version, at https://doi.org/10.31083/j.rcm2308263.
