# Supplementary material for: Triglyceride-Glucose Index Linked to In-Hospital Mortality in Critically Ill Patients with Heart Disease
Source: Rev Cardiovasc Med. 2022 Jul 21;23(8):263. doi: 10.31083/j.rcm2308263 (PMC11266948; doi:10.31083/j.rcm2308263)
Supplement: Supplementary file 1 [file 2153-8174-23-8-263-s1.docx]

*Original Research*

Supplementary material: Triglyceride-Glucose Index Linked to In-Hospital Mortality in Critically Ill Patients with Heart Disease

Guangyao Zhai^1^, Jianlong Wang^1^, Yuyang Liu^1^, Yujie Zhou^1,^*

^1^Beijing AnZhen Hospital: Capital Medical University Affiliated Anzhen Hospital, 100089 Beijing, China

*Correspondence: [azzyj12@163.com](mailto:azzyj12@163.com) (Yujie Zhou)

**Data extraction:**

Following data were collected: demographics (age, gender and race), vital signs (blood pressure, heart rate, respiration rate, oxygen saturation), body mass index, diagnoses and comorbidities (congestive heart failure, coronary artery disease, acute coronary syndrome, ST-elevation myocardial infarction(STEMI), non-ST-elevation myocardial infarction(NSTEMI), arrhythmias, cardiac arrest, bradycardia, atrial fibrillation, ventricular arrhythmias, atrioventricular block, cardiomyopathy, valve disease, shock, pulmonary embolism, pulmonary hypertension, hypertension, diabetes, hypercholesterolemia, chronic obstructive pulmonary disease(COPD), respiratory failure, chronic kidney disease, acute kidney injury, malignancy, stroke, sepsis), laboratory parameters(white blood cell, lymphocyte, monocyte and neutrophil percentage, red blood cell platelet, hemoglobin, hematocrit, glucose, triglyceride, creatinine, blood nitrogen urea, sodium, potassium), medication use( antiplatelet, oral anticoagulants, beta-blockers, angiotensin-converting enzyme inhibitor/angiotensin receptor blocker(ACEI/ARB), statin), acute physiology score(APS) and Acute Physiology and Chronic Health Evaluation IV (APACHE IV)
